# *WRKY* Genes Improve Drought Tolerance in *Arachis duranensis*

**DOI:** 10.3389/fpls.2022.910408

**Published:** 2022-05-26

**Authors:** Yongli Zhang, Pei Du, Faqian Xiong, Xiaojun Zhang, Hui Song

**Affiliations:** ^1^Grassland Agri-Husbandry Research Center, College of Grassland Science, Qingdao Agricultural University, Qingdao, China; ^2^Industrial Crops Research Institute, Henan Academy of Agricultural Sciences/Key Laboratory of Oil Crops in Huang-Huai-Hai Plains, Ministry of Agriculture and Rural Affairs/Henan Provincial Key Laboratory for Oil Crops Improvement, Zhengzhou, China; ^3^Cash Crops Research Institute, Guangxi Academy of Agricultural Sciences, Nanning, China; ^4^College of Agronomy, Qingdao Agricultural University, Qingdao, China

**Keywords:** *Arachis duranensis*, drought tolerance, protein–protein interaction, regulatory network, WRKY

## Abstract

WRKY transcription factor participates in plant growth and development and response to biotic and abiotic stresses. *Arachis duranensis*, a turfgrass, has high drought tolerance, yet little is known about *AdWRKYs* response to drought stress in *A. duranensis*. In this study, RNA-seq identified five *AdWRKYs*, including *AdWRKY18*, *AdWRKY40*, *AdWRKY42*, *AdWRKY56*, and *AdWRKY64*, which were upregulated under drought stress. Orthologous relationships between *AdWRKYs* and *Arabidopsis WRKY* were determined to predict the regulatory networks of the five AdWRKYs based on AtWRKYs. Additionally, protein–protein interactions were predicted using differentially expressed proteins from RNA-seq. The quantitative real-time PCR (qRT-PCR) results showed that *AdWRKY40* was upregulated, while *AdWRKY42*, *AdWRKY56*, and *AdWRKY64* were downregulated at different time-points under drought stress. The predicted regulatory networks showed that AdWRKY40 activates *COR47*, *RD21*, and *RD29A* expression under drought stress. Besides, AdWRKY56 regulated *CesA8* under drought stress. Aradu.YIQ80 (NAC019) interacted with AdWRKY40, AdWRKY42, AdWRKY56, and AdWRKY64, while Aradu.Z5H58 (NAC055) interacted with AdWRKY42 and AdWRKY64 under drought stress. This study used *Arabidopsis* to assess *AdWRKYs* function and regulatory networks, providing a basis for understanding drought tolerance in *A. duranensis*.

## Introduction

Drought severely impairs plant growth and development (Zhu, 2002, 2016). Therefore, plants have evolved complex adaptive strategies to cope with drought stress over time. Plants reduce water loss by regulating stomatal aperture and root development (Zhu, 2016; Ahammed et al., 2020). Briefly, accumulated abscisic acid (ABA) content changes the Ca^2+^ concentration of the guard cell, which activates Ca^2+^ signaling to increase water loss resulting in stomatal closure under drought stress (MacRobbie, 2006). High ABA content reduces the germination rate, root development, and plant growth (Finkelstein et al., 2002; Fujita et al., 2011). Similarly, accumulated ROS activates Ca^2+^ and K^+^ signaling, promoting stomatal closure under drought stress (Livak and Schmittgen, 2001; Sierla et al., 2016; Qi et al., 2018). Besides, plants also scavenge reactive oxygen species (ROS) to prevent harm (Zhu, 2016; Ahammed et al., 2020). Under drought stress, excess ROS content causes lipid peroxidation, protein degradation, nucleotide damage, cell death, and electron transport chain damage (Sierla et al., 2016; Xu et al., 2016; Qi et al., 2018). Oxidases (SOD, POD, and CAT) and non-oxidases (APX, GSH, and AsA) can scavenge ROS in plants (Sierla et al., 2016; Qi et al., 2018).

The WRKY transcription factor is involved in drought stress response (Rushton et al., 2010, 2012; Chen et al., 2017a). Besides the one conserved heptapeptide WRKYGQK motif located on the N-terminal WRKY domain (Eulgem et al., 2000), WRKY contains two types of zinc-finger structure, C-X_4-5_-C-X_22-23_-H-X-H (C_2_H_2_) and C-X_5-8_-C-X_25-28_-H-X_1-2_-C (C_2_HC; Eulgem et al., 2000; Rushton et al., 2010). WRKY can be classified into three groups based on WRKY domain number and zinc-finger structure type. Group I contains two WRKY domains and a C_2_H_2_; Group II contains one WRKY domain and a C_2_H_2_; and Group III contains one WRKY domain and a C_2_HC (Eulgem et al., 2000; Rushton et al., 2010). *WRKYs* have been identified at the genome level due to the development of sequencing technology: *Arabidopsis thaliana* (Eulgem et al., 2000), *Oryza sativa* (Ross et al., 2007), *Medicago truncatula* (Song and Nan, 2014), *Triticum aestivum* (Gupta et al., 2018), *Glycine max* (Song et al., 2016a), and *Arachis* species (Song et al., 2016b; Zhao et al., 2020). However, the role of *WRKY* has not been widely studied in *Arachis*.

WRKY controls gene expression by binding to the W-box element (C/TTGACT/C) of downstream genes (Eulgem et al., 2000; Ciolkowski et al., 2008; Rushton et al., 2010). Extensive studies have also demonstrated that *WRKY* improves drought stress response (Phukan et al., 2016; Jiang et al., 2017; Chen et al., 2017a, 2019). Moreover, *AtWRKY11* and *AtWRKY17* overexpression improves drought tolerance in *Arabidopsis*, promoting seed germination and root growth under drought stress (Ali et al., 2018). *OsWRKY11* enhances drought tolerance in rice by upregulating *HSP101 expression* (Wu et al., 2009). WRKY regulates drought stress through abscisic acid (ABA) signaling (Rushton et al., 2012). AtWRKY57 improves drought stress in *Arabidopsis* by binding their W-box elements to activate the expressions of *RD29A* and *NCED3* (Jiang et al., 2012). TaWRKY2 increases the drought and salt tolerance of wheat by binding to the W-box of *STZ* and *RD29B* (Niu et al., 2012). TaWRKY19 also increases drought and salt tolerance of wheat by binding to W-box of *DREB2A* and *Cor6.6*, thus activating *DREB2A*, *RD29A*, *DR29B*, and *Cor6.6* (Niu et al., 2012). GmWRKY54 enhances drought stress of soybean by activating *PYL8*, *SRK2A*, *CIPK11*, and *CPK3* (Wei et al., 2019). WRKY53 enhances drought stress of *Pyrus betulaefolia* by binding to the W-box of *PbrNCED1* (Liu et al., 2019). Therefore, different plants regulate various downstream genes involved in drought stress tolerance.

*Arachis duranensis*, a turfgrass, has strong drought tolerance (Leal-Bertioli et al., 2012, 2018). The genome sequences of *A. duranensis* and drought-related RNA-seq datasets are available in the PeanutBase database (Brasileiro et al., 2015; Bertioli et al., 2016; Dash et al., 2016). A previous study identified 75 WRKYs (AdWRKYs) in *A. duranensis* (Song et al., 2016b), providing a basis for identifying *AdWRKYs* involved in drought stress response. In this RNA-seq based study, five *AdWRKYs* (*AdWRKY18*, *AdWRKY40*, *AdWRKY42*, *AdWRKY56*, and *AdWRKY64*) were differentially expressed under drought stress. The regulatory networks of AdWRKYs were then determined and verified. This work provides a theoretical basis for further analysis of the function of *AdWRKYs*.

## Materials and Methods

### Identification of *AdWRKYs* Involved in Drought Stress Response

The transcriptomes of *A. duranensis* under drought stress and normal growth conditions were sequenced and *de novo* assembled to detect differentially expressed genes (DEGs) under normal growth and drought stress conditions in 2015 (Brasileiro et al., 2015). In 2016, the RNA-seq was re-assembled using the *A. duranensis* genome as the reference, and the updated RNA-seq data were released in the PeanutBase database (Dash et al., 2016). The DEGs were identified between drought and control using the edgeR program (Robinson et al., 2010). Genes with log_2_(Foldchange) > 2 or <−2 at FDR < 0.05 were considered differentially expressed (Brasileiro et al., 2015; Dash et al., 2016).

A previous study identified WRKYs in *A. duranensis* (Song et al., 2016b). This study extracted the differentially expressed *AdWRKY* genes in the abovementioned RNA-seq datasets. The differentially expressed *AdWRKY* features and subcellular localization were predicted using ExPASy (Gasteiger et al., 2003) and Plant-mPLoc (Chou and Shen, 2010) with default parameters.

### Phylogenetic Relationship Analysis

Phylogenetic trees were constructed using AdWRKYs and Arabidopsis WRKY (AtWRKY) to reveal their orthologous relationship. AtWRKYs were obtained from a public database (The *Arabidopsis* Information Resource, https://www.arabidopsis.org/index.jsp). Multiple sequence alignments were conducted using the MAFFT program (Katoh and Standley, 2013). The ProtTest program was used to estimate the best-fit model for constructing phylogenetic trees (Darriba et al., 2011). The maximum likelihood (ML) of the trees was determined using the IQ-tree program (Nguyen et al., 2015).

### *Cis*-Acting Element Analysis

WRKY regulates downstream gene expression by binding to *cis*-acting elements, such as W-box (C/TTGACC/T), WT-box (GACTTT), WK-box (TTTTCCAC), PRE (TACTGCGCTTAGT), and SURE (TAAAGA TTACTAATAGGAA; Rinerson et al., 2015; Chen et al., 2019). Herein, the TBtools program was used to extract the 2-kb sequences upstream of the start codon of the predicted genes to identify AdWRKYs genes involved in regulation (Chen et al., 2020). PlantCARE (Rombauts et al., 1999) was used to predict the binding sites of the WRKY transcription factor.

### Protein Interaction Analysis

WRKYs interact with other proteins involved in plant development and stress response (Hu et al., 2013; Chen et al., 2017b; Zhang et al., 2018). The protein interaction of AdWRKYs and their differentially expressed genes were predicted using the STRING public database^1^ with *Arabidopsis* protein sequences as the reference.

### Quantitative Real-Time PCR Analyses

Quantitative real-time PCR (qRT-PCR) analyses were used to verify the drought-tolerance function of the abovementioned genes. Briefly, the *A. duranensis* seeds were sterilized and germinated on wet filter paper at 28°C. The seedlings were then transferred to the Hoagland solution. Four-leaf plants were treated with 10% (w/v) PEG6000. The leaves were collected after 0, 6, 12, 24, 36, and 48 h of treatments. The control was sampled from 0 h. Three biological replicates were used.

Plant RNA Extraction Kit (TaKaRa, Dalian, China) was used to extract total RNA. The RNA (1 μg) was used for cDNAs synthesis *via* Reverse Transcriptase M-MLV System (TaKaRa, Dalian, China). The primers were designed by Beacon Designer 8 (Supplementary Table S1). The primers were specifically for amplification since WRKY sequences are conserved (Zhang et al., 2020a). qRT-PCR was performed using TB green premix ex Taq II (TaKaRa, Dalian, China) on the CFX96 real-time PCR machine (Bio-Rad, CA, United States) with UBI2 as the reference gene (Morgante et al., 2011). The PCR conditions included: 95°C denaturation for 30 s, followed by 40 cycles at 95°C for 5 s and 60°C for 45 s. A melting curve analysis was performed at the end of the PCR running end. The 2^-ΔΔCt^ method was used for quantification (Livak and Schmittgen, 2001).

## Results and Discussion

### *AdWRKYs* Tolerant to Drought Stress

**Figure 1:**
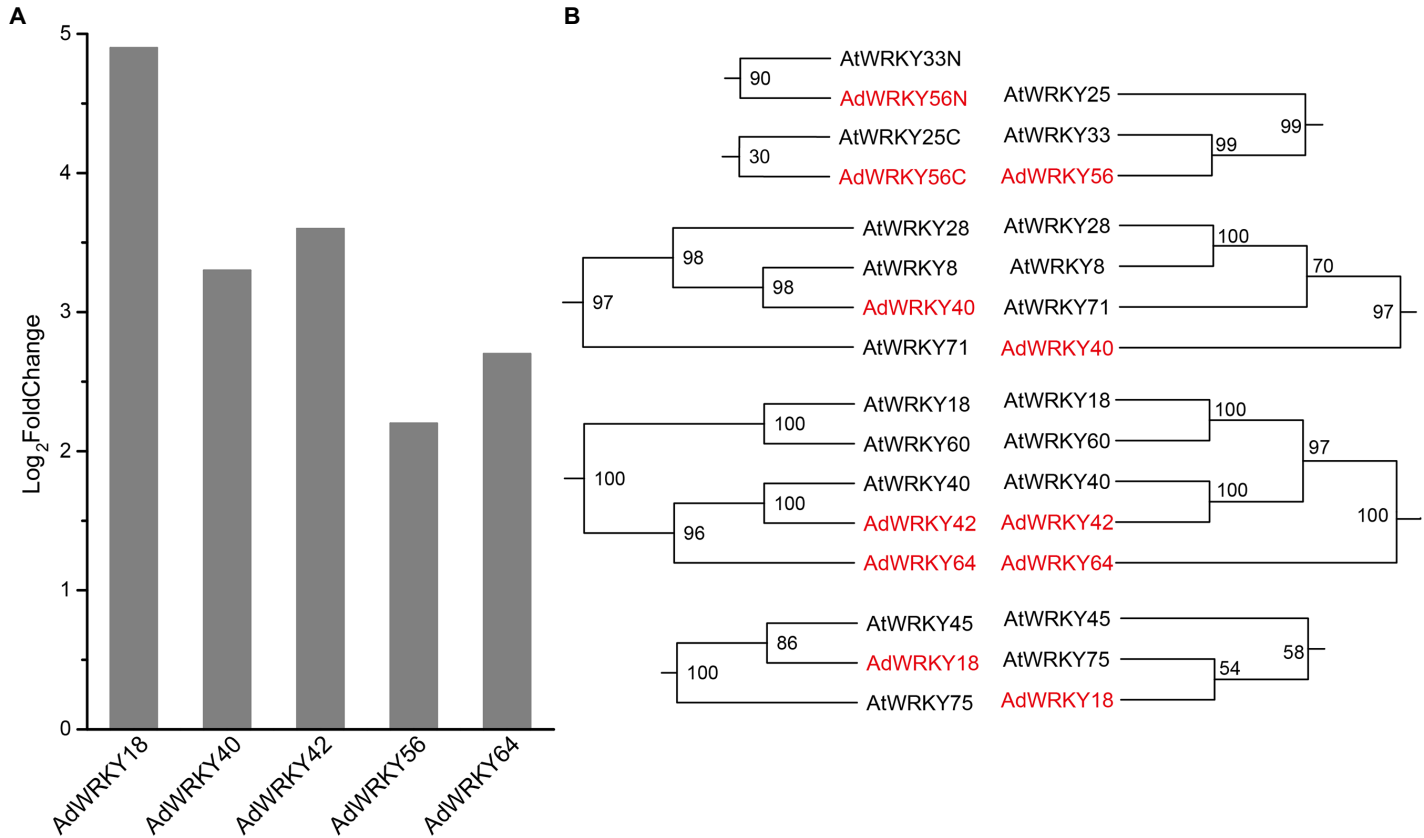
Differentially expressed *AdWRKYs* and phylogenetic trees. **(A)** The differential gene expression based on RNA-seq (Brasileiro et al., 2015). **(B)** The maximum likelihood trees construct using WRKY domains and full-length proteins.

Five *AdWRKYs* were identified in *A. duranensis* RNA-seq datasets. These *AdWRKYs* (*AdWRKY18*, *AdWRKY40*, *AdWRKY42*, *AdWRKY56*, and *AdWRKY64*) had full-length sequences and were upregulated (Figure 1A). *AdWRKY18* and *AdWRKY56* had the highest (Log_2_foldchange = 4.9; Figure 1A), and lowest differential expression (Log_2_foldchange = 2.2, Figure 1A), respectively. The CDS length, DNA length, isoelectric point, and molecular weight were 471–1,623 bp, 783–2,328 bp, 6.59–9.67, and 18364.62–59830.41 Da (Table 1), respectively. The AdWRKYs were predicted in location in the nucleus (Table 1).

**Table 1:** *AdWRKYs* features.

Gene name	*Arachis* ID	CDS length (bp)	Gene length (bp)	Isoelectric point	Molecular weight (Da)	Subcellular localization
*AdWRKY18*	Aradu.B1C6F	471	783	9.67	18364.62	Nucleus
*AdWRKY40*	Aradu.I8GKQ	1,098	1,098	6.78	40064.95	Nucleus
*AdWRKY42*	Aradu.KEE43	963	1874	8.31	35677.08	Nucleus
*AdWRKY56*	Aradu.S7YD6	1,623	2,328	6.59	59830.41	Nucleus
*AdWRKY64*	Aradu.VE705	879	879	6.92	32729.67	Nucleus

The ML trees were constructed using WRKY domains and WRKY full-length proteins to reveal the orthologous relationships between *AdWRKYs* and *AtWRKYs*. The best-fit models were JTT + I + G and VT + I + G + F. The phylogenetic tree showed that AdWRKY42 had homology with AtWRKY40 (Figure 1B; Supplementary Figures S1, S2). The other four AdWRKYs had different topological structures between the WRKY domain tree and the WRKY full-length protein tree (Figure 1B; Supplementary Figures S1, S2). Altogether, the following homologous relationships were obtained based on two ML trees: AdWRKY18 with AtWRKY45 and AtWRKY75; AdWRKY40 with AtWRKY8, AtWRKY28, and AtWRKY71; AdWRKY42 with AtWRKY40; AdWRKY56 with AtWRKY33 and AtWRKY25; and AdWRKY64 with AdWRKY42, AtWRKY40, AtWRKY60, and AtWRKY18.

Experiments have shown the functions of many *AtWRKYs*. *AtWRKY45* overexpression alleviates phosphate starvation and promotes leaf senescence in *Arabidopsis* (Wang et al., 2014; Chen et al., 2017b). Overexpression of *AtWRKY75* promotes leaf senescence and flowering in *Arabidopsis* (Guo et al., 2017; Zhang et al., 2018). A recent study showed that *PtrWRKY75*, which is orthologous with *AtWRKY75*, improves drought tolerance in polar (Zhang et al., 2020b), indicating that *AdWRKY18* may confer drought tolerance traits.

Overexpression of *AtWRKY8* improves salt stress (Hu et al., 2013). Besides, *AtWRKY28* and *AtbHLH17* overexpression enhance drought and salt tolerance in *Arabidopsis* (Babitha et al., 2013). These results indicate that *AdWRKY40* is tolerant to drought and salt stresses.

Drought stress induces *AtWRKY40* expression (Chen et al., 2010), consistent with *A. duranensis* RNA-seq dataset results, which showed that *AdWRKY42* is upregulated under drought stress. *AtWRKY18*, *AtWRKY40*, and *AtWRKY60* are involved in the ABA-signaling pathway, but *AtWRKY40* antagonizes *AtWRKY18* and *AtWRKY60* functions (Chen et al., 2010; Shang et al., 2010). Herein, *AdWRKY64* had homology with *AtWRKY18*, *AtWRKY40*, and *AtWRKY60*, indicating that *AdWRKY64* is involved in drought stress response.

Overexpression of *AtWRKY33* enhances drought tolerance in *Arabidopsis* (Kim et al., 2013; Wang et al., 2013), indicating that drought stress induces *AdWRKY56*.

Therefore, *AdWRKY18*, *AdWRKY40*, *AdWRKY42*, *AdWRKY56*, and *AdWRKY64* have potential functions in drought stress response based on the abovementioned homologous relationships.

### AdWRKYs Improve Drought Tolerance by Regulating Downstream Genes

**Figure 2:**
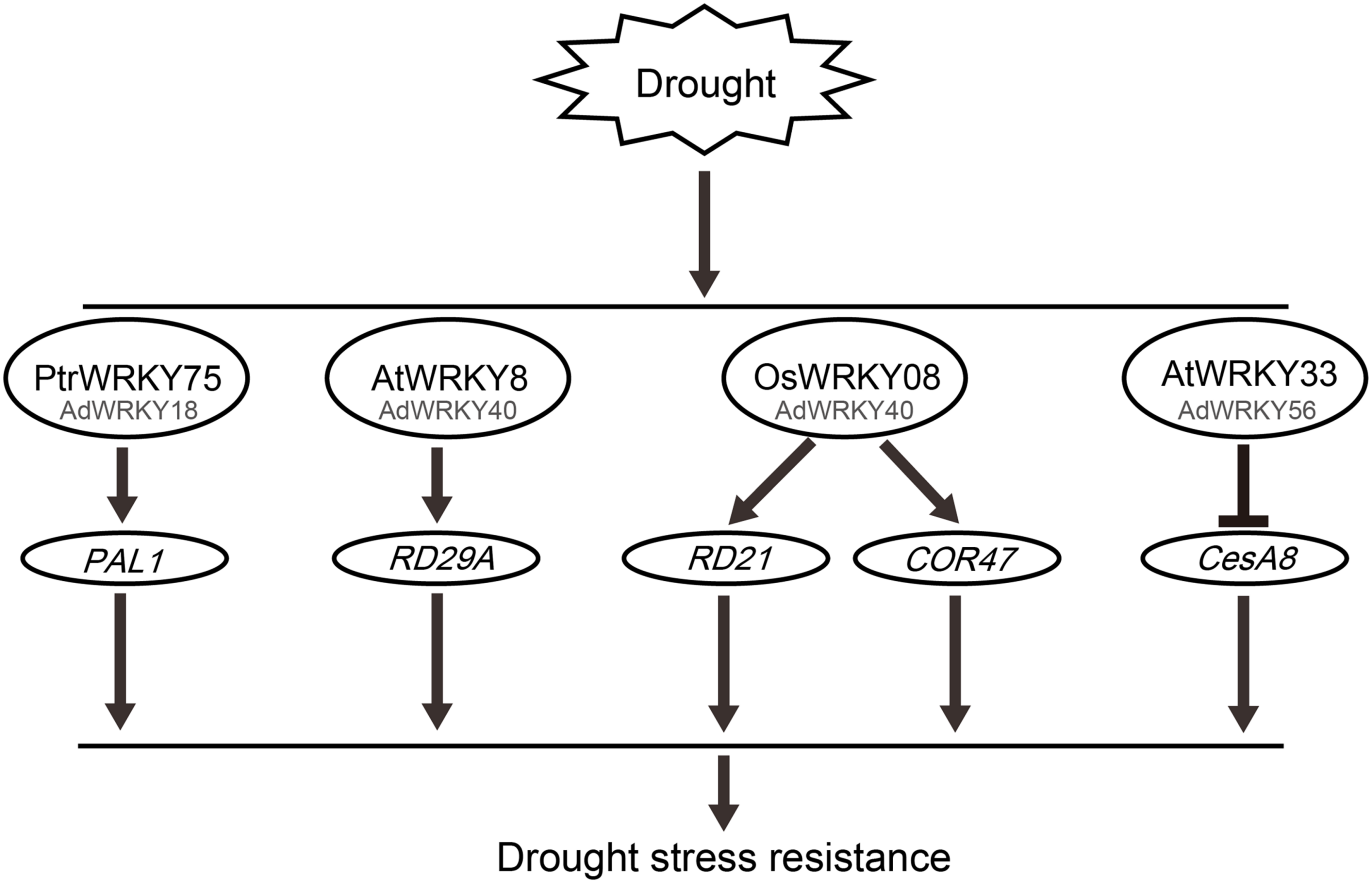
Potential roles of AdWRKYs regulate downstream genes under drought stress. The datasets are obtained from previous studies, including PtrWRKY75 (Zhang et al., 2020b), AtWRKY8 (Hu et al., 2013), OSWRKY8 (Song et al., 2010), and AtWRKY33 (Wang et al., 2013). The WRKY activates PAL1, RD29A, RD21, and COR47 genes expression, and represses CesA8 gene expression.

*AdWRKY18* has homology with *PtrWRKY75*. *PtrWRKY75* directly regulates PHENYLALANINE AMMONIA LYASE 1 (*PAL1*; Figure 2), involved in the salicylic acid (SA) pathway, to scavenge ROS, thus improving drought stress (Zhang et al., 2020b).

*AdWRKY40* had homology with *AtWRKY8*, *AtWRKY28*, and *AtWRKY71*. AtWRKY8 enhances salt tolerance by activating *AtRD29A* expression (Hu et al., 2013). Drought, salt, and ABA induce *RD29A* expression (Narusaka et al., 2003; Hua et al., 2006). These results indicate that AtWRKY8 increases drought tolerance by activating *AtRD29A*. *OsWRKY08* has homology with *AtWRKY28* and *AtWRKY71* (Song et al., 2010). OsWRKY08 improves drought stress by inducing *RD21* and *COR47* expression (Song et al., 2010).

Herein, *AdWRKY42* had homology with *AtWRKY40*. Although drought stress induces *AtWRKY40* expression (Chen et al., 2010), it is unknown how *AtWRKY40* regulates drought stress. *CRK5* improves drought tolerance (Lu et al., 2016). Although *AtWRKY18*, *AtWRKY40*, and *AtWRKY60* triple mutants inhibit *CRK5* expression, *AtWRKY40* antagonizes *AtWRKY18* and *AtWRKY60* (Shang et al., 2010; Lu et al., 2016), indicating that only AtWRKY40 does not regulate drought stress through *CRK5*.

*AtCesA8*, a cellulose synthase catalytic subunit, plays a crucial role in cellulose synthesis in the secondary cell wall (Taylor et al., 2000, 2003). *AtCesA8* mutant accumulates high ABA content, thus reducing the expression of stress-related genes (Chen et al., 2005). *AtCesA8* negatively regulates drought stress (Chen et al., 2005; Wang et al., 2013). AtWRKY33 decreases gene expression by binding to *CesA8* W-box element, thus increasing drought tolerance (Wang et al., 2013). Herein, AdWRKY56 had homology with AtWRKY33, indicating that AdWRKY56 can enhance drought tolerance by controlling *CesA8*. Therefore, these results suggest that AdWRKY18, AdWRKY40, and AdWRKY56 improve drought tolerance by regulating the expression of downstream genes.

WRKYs control downstream genes by binding to W-box, WT-box, WK-box, PRE, and SUR *cis*-acting elements (Sun et al., 2003; van Verk et al., 2008; Hu et al., 2013; Xiao et al., 2013; Machens et al., 2014; Rinerson et al., 2015; Chen et al., 2019). The *AtPAL1*, *AtRD29A*, *AtRD21*, *AtCOR47*, and *AtCesA8* are orthologous with *Aradu.NNP8F*, *Aradu.MEI7N*, *Aradu.08WSJ*, *Aradu.IF4XP*, and *Aradu.UPY7V* in *A. duranensis*. *Aradu.NNP8F*, *Aradu.MEI7N*, and *Aradu.08WSJ* lack W-box element in the 2-kb promote region (Supplementary Table S2). AtWRKY33 inhibits *CesA8* expression by binding to the distal W-box (~3-kb) of the *CesA8* gene, thus increasing drought tolerance (Wang et al., 2013). The 3-kb promoter region of *Aradu.NNP8F*, *Aradu.MEI7N*, and *Aradu.08WSJ* also lack the W-box elements (Supplementary Table S2). These results indicate that AdWRKY18 cannot directly regulate *Aradu.NNP8F* (*PAL1*) under drought stress and AdWRKY40 cannot directly regulate *Aradu.08WSJ* (*RD21*) and *Aradu.MEI7N* (*RD29A*) under drought stress. Notably, RNA-seq showed that *Aradu.IF4XP* (*COR47*) gene was differentially expressed after drought stress, indicating that AdWRKY40 potentially regulates *Aradu.IF4XP* by binding the W-box element under drought stress.

Subcellular localization showed that AtPAL1 and Aradu.NNP8F are located in the cytoplasm, AtRD21 and Aradu.08WSJ are located in the vacuole, AtCesA8 and Aradu.UPY7V are located in the chloroplast, and others are located in the nucleus (Supplementary Table S2). A previous study revealed that AtWRKY40 moves from the nucleus to the cytoplasm to control downstream genes (Rushton et al., 2012). Similarly, AdWRKYs can potentially move from the nucleus to other organelles to regulate Aradu.NNP8F, Aradu.08WSJ, and Aradu.UPY7V is located outside the nucleus.

### AdWRKYs Improve Drought Tolerance Through Protein–Protein Interactions

**Figure 3:**
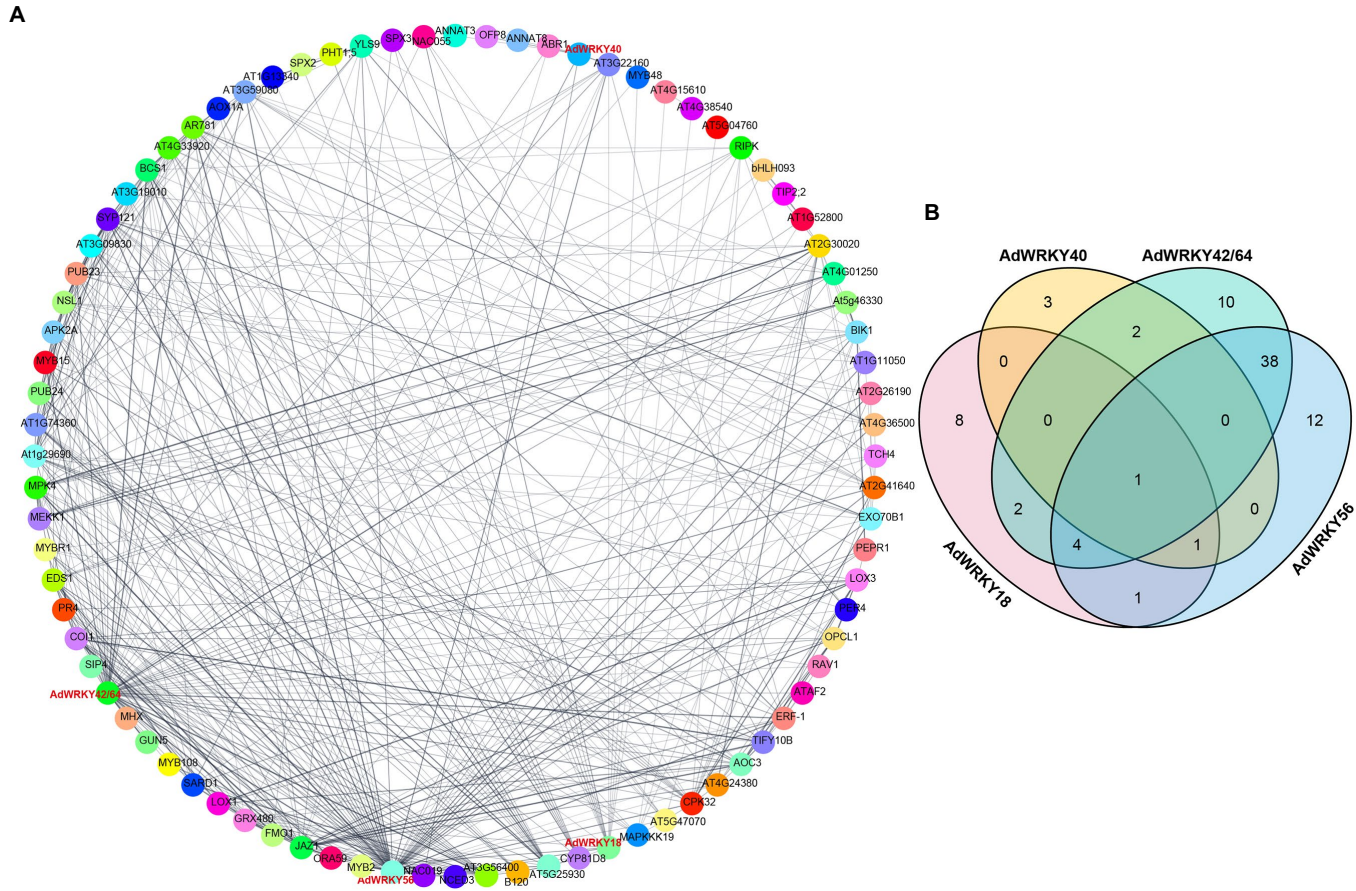
The interaction between AdWRKYs and other proteins. **(A)** The interaction between AdWRKYs and other proteins. **(B)** The number of other proteins interacting with AdWRKYs.

WRKYs can interact with other proteins involved in drought stress regulation (Perruc et al., 2004; Cheng et al., 2012; Hu et al., 2013). Herein, AdWRKY42 and AdWRKY64 mapped on the same *Arabidopsis* protein sequence, AT1G80840. AdWRKY18, AdWRKY40, AdWRKY42, AdWRKY56, and AdWRKY64 directly interacted with 17, 7, 57, 57, and 57 proteins, respectively (Figure 3A; Supplementary Table S3).

**Figure 4:**
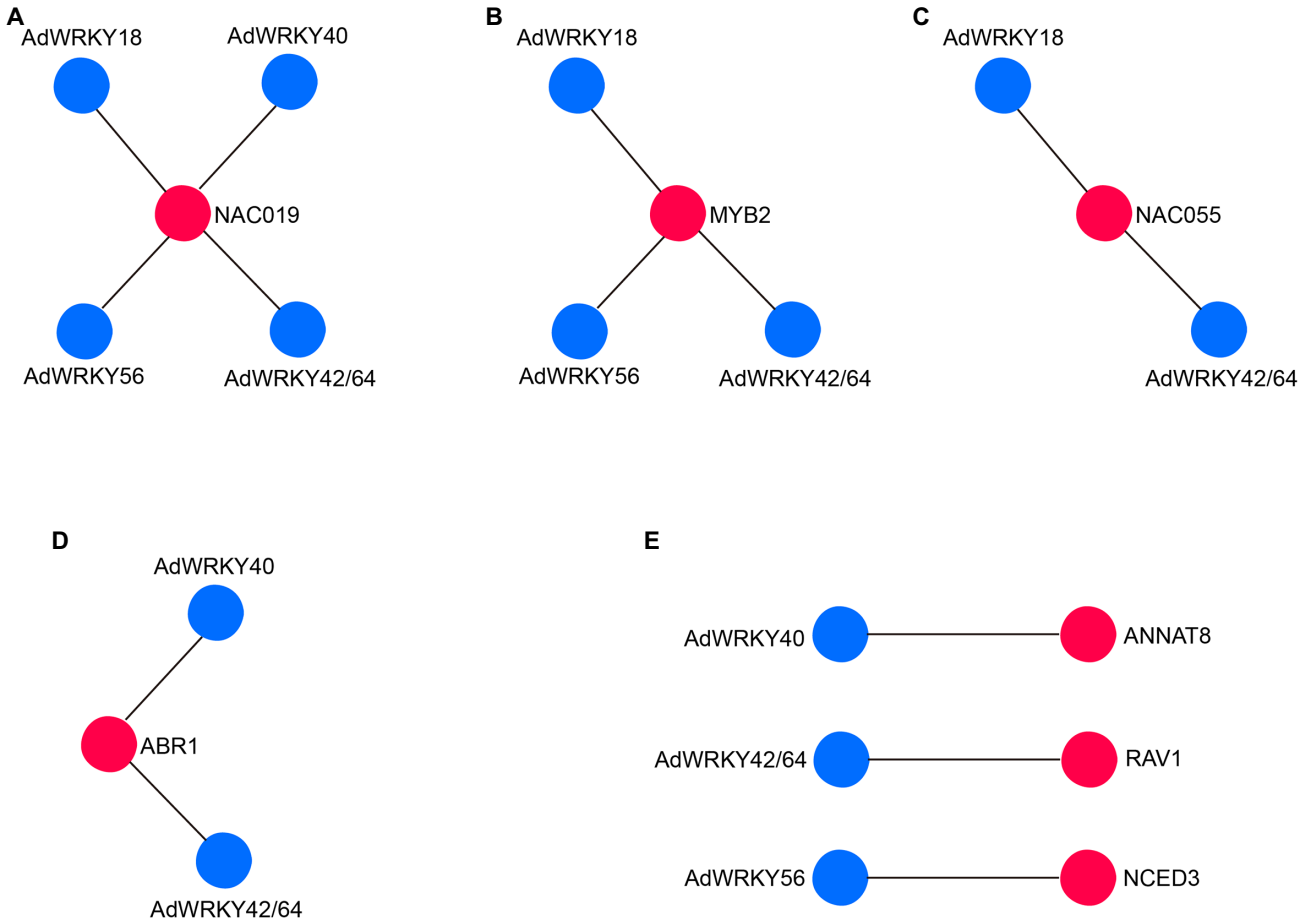
AdWRKYs interaction with proteins with drought-tolerance function based on *Arabidopsis*. **(A)** NAC019 interaction with AdWRKYs. **(B)** MYB2 interaction with AdWRKYs. **(C)** NAC055 interaction with AdWRKYs. **(D)** ABR1 interaction with AdWRKYs. **(E)** Interaction between AdWRKYs and other specific proteins.

The five AdWRKYs interacted with common proteins and specific proteins (Figure 3B), of which four common proteins (NAC019, MYB2, NAC055, and ABR1) are involved in drought stress response. Aradu.YIQ80 (NAC019) interacted with the five AdWRKYs (Figure 4A; Supplementary Table S4) while Aradu.X7LBF (MYB2), Aradu.Z5H58 (NAC055), and Aradu.ME4LN (ABR1) interacted with at least two AdWRKYs (Figures 4B–D; Supplementary Table S4). AtNAC019 improves drought stress in *Arabidopsis* by activating *ERD1* expression (Tran et al., 2004). AtWRKY1 enhances drought stress or ABA treatment by binding to the W-box *cis*-acting element of *AtMYB2* (Qiao et al., 2016). *AtNAC055* overexpression increases drought tolerance, and the *AtNAC055* mutant has a decreased drought tolerance (Fu et al., 2018). Drought stress induces *AtABR1* expression. However, mannitol stress can decrease *AtABR1* expression, thus, reducing seed germination (Pandey et al., 2005).

AdWRKY40, AdWRKY42/64, and AdWRKY56-specific interaction with Aradu.MBZ2M (ANNAT8), Aradu.7AQ1B (RAV1), and Aradu.FJ7R7 (NCED3) are involved in drought stress response (Figure 4E; Supplementary Table S5). Heterologous expression of *ANNAT8* enhances the response to drought and salt stresses in *Arabidopsis* and tobacco during the growth and development stages (Yadav et al., 2016). Overexpression of *RAV1 promotes* water loss by activating the expression of ABA-responsive genes (Fu et al., 2014), indicating that *RAV1* negatively regulates drought stress. *CED3* responds to water deficit through ABA synthesis (Sato et al., 2018; Baek et al., 2020).

### Verification of Drought Tolerance of AdWRKYs and Their Regulatory Genes Using qRT-PCR

**Figure 5:**
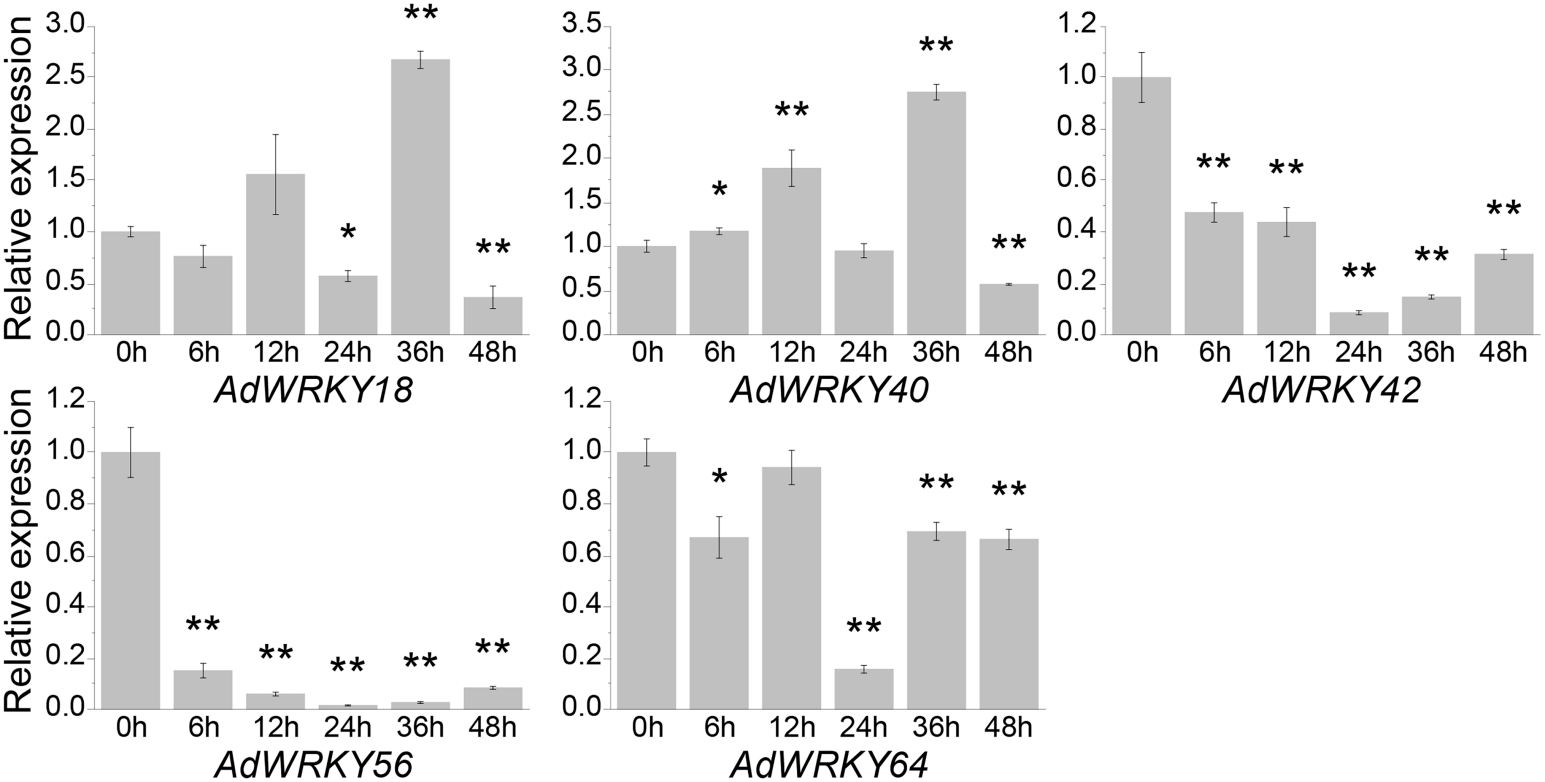
Differentially expressed *AdWRKYs* under drought stress detected by quantitative real-time PCR (qRT-PCR). Four-leaf plants were treated with 10% (w/v) PEG6000. The leaves were collected after 0, 6, 12, 24, 36, and 48 h of treatments. The control was sampled from 0 h. Three biological replicates were used. Asterisks * and ** indicate significant differences at 0.05 and 0.01 using *t* tests, respectively.

This study identified five differentially expressed *AdWRKYs* using RNA-seq and their regulatory networks based on *Arabidopsis*. qRT-PCR was used to assess the drought stress response of the five *AdWRKYs* and their regulatory genes. *AdWRKY42*, *AdWRKY56*, and *AdWRKY64* genes were downregulated at 6, 12, 24, 36, and 48 h (Figure 5). *AdWRKY40* was upregulated at 6, 12, and 36 h, and downregulated at 48 h (Figure 5). *AdWRKY18* was downregulated at 24 and 48 h and upregulated at 36 h (Figure 5). The five *AdWRKYs* were downregulated at 48 h. *AdWRKY40* had similar qRT-PCR and RNA-seq results. The differential expression of *AdWRKY42*, *AdWRKY56*, and *AdWRKY64* contradicted the RNA-seq results. The possible reason is different drought treatment and cultural environments between qRT-PCR and RNA-seq. The RNA-seq data were produced from the samples under natural drought and normal growth conditions (Brasileiro et al., 2015), while qRT-PCR was analyzed under five drought-stress time points using 10% (w/v) PEG6000 treatments.

**Figure 6:**
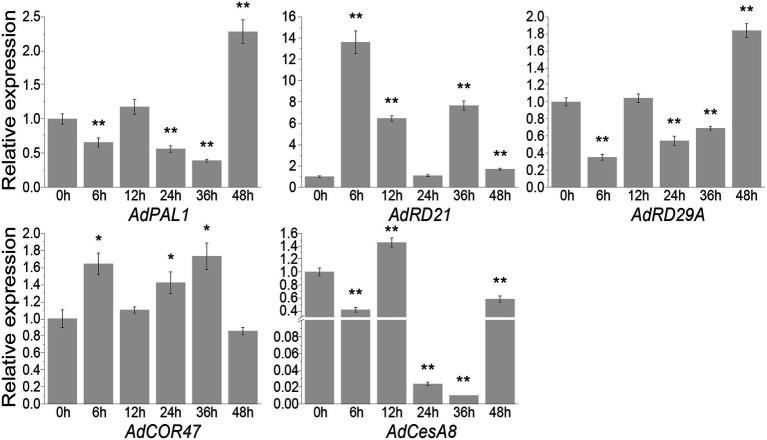
Differential expression levels of AdWRKYs regulating downstream genes under drought stress, assessed using quantitative real-time PCR. Four-leaf plants were treated with 10% (w/v) PEG6000. The leaves were collected after 0, 6, 12, 24, 36, and 48 h of treatments. The control was sampled from 0 h. Three biological replicates were used. Asterisks * and ** indicate significant differences at 0.05 and 0.01 using *t* tests, respectively.

Quantitative real-time PCR showed that *AdCOR47* and *AdCesA8* genes were upregulated and downregulated, respectively, at five time-points (Figure 6). Similarly, previous studies showed that *COR47* is positively regulated in *Oryza sativa* under drought stress, while *CesA8* is negatively regulated in *A. thaliana* under drought stress (Song et al., 2010; Wang et al., 2013). Herein, *AdPAL1* and *AdRD29A* were downregulated at 6, 12, 24, and 36 h (Figure 6). However, *PtrPAL1* and *AtRD29A* are positively regulated under drought stress (Hu et al., 2013; Zhang et al., 2020b). WRKYs directly regulate *PtrPAL1* and *RD29A* by binding to W-box elements. Although *AdPAL1* and *AdRD29A* do not have W-box elements, they were upregulated at 48 h (Figure 6). *AdRD21* also lacked the W-box element and was upregulated at five time-points. Similarly, a previous study showed that *AtRD21* is positively regulated under drought stress (Narusaka et al., 2003; Hua et al., 2006; Song et al., 2010). Therefore, AdWRKY40 is involved in regulating different regulatory networks of downstream genes *AdRD21* and *AdRD29A*.

**Figure 7:**
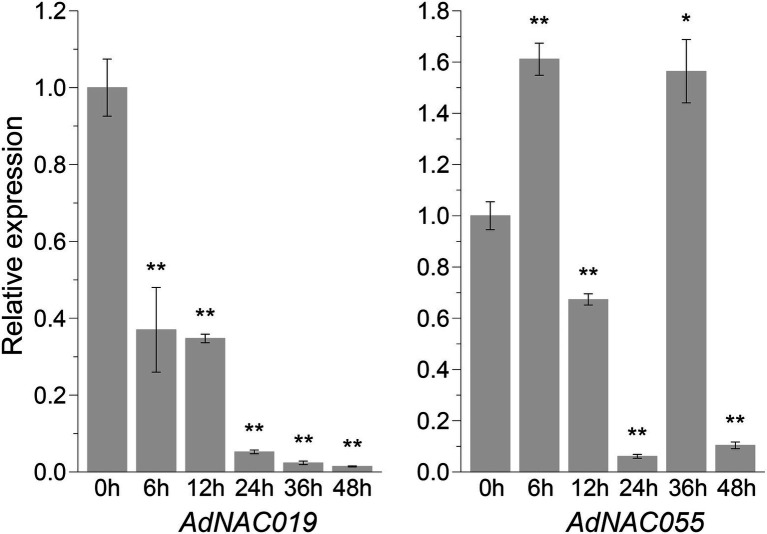
Differential expression levels of genes that are translated proteins interaction with AdWRKYs under drought stress detected *via* quantitative real-time PCR. Four-leaf plants were treated with 10% (w/v) PEG6000. The leaves were collected after 0, 6, 12, 24, 36, and 48 h of treatments. The control was sampled from 0 h. Three biological replicates were used. Asterisks * and ** indicate significant differences at 0.05 and 0.01 using *t* tests, respectively.

Quantitative real-time PCR was used to assess the drought stress response of the genes that translated proteins-AdWRKYs interaction. This study found that *AdNAC019* and *AdNAC055* were differentially expressed after drought stress. However, other genes were not differentially expressed relative to the control group. AdNAC019 interacted with the five AdWRKYs, while AdNAC055 interacted with only AdWRKY42 and AdWRKY64. *AdNAC019* was downregulated at the five time-points (Figure 6). *AdNAC055* was upregulated at 6 and 36 h and downregulated at 12, 24, and 48 h (Figure 7). However, NAC019 and NAC055 positively regulate drought stress in *Arabidopsis* (Tran et al., 2004; Fu et al., 2018). Similarly, AdWRKY42, AdWRKY56, and AdWRKY64 were downregulated after drought stress, different from the expression patterns in *Arabidopsis*. These results indicate that *Arachis* and *Arabidopsis* have different regulatory networks under drought stress.

### Opportunities and Challenges in AdWRKYs Study

Herein, five *AdWRKYs* were identified under drought stress based on previous studies and RNA-seq (Brasileiro et al., 2015; Song et al., 2016b). Subsequently, orthologous relationships between AdWRKYs and AtWRKYs were constructed. The regulatory networks of the five AdWRKYs were determined based on AtWRKYs and verified using qRT-PCR. The results showed that AdWRKY40 positively regulated drought stress, while AdWRKY42, AdWRKY56, and AdWRKY64 negatively regulated drought stress. Moreover, AdWRKY40 was upregulated under drought stress, confirming the orthologous WRKYs from *Arabidopsis* and *Oryza* (Narusaka et al., 2003; Hua et al., 2006; Song et al., 2010; Hu et al., 2013). However, the orthologous AdWRKY42, AdWRKY56, and AdWRKY64 showed the opposite results in *Arabidopsis* under drought stress. Besides, qRT-PCR and RNA-seq results showed opposite results in *A. duranensis*. Additionally, *Arabidopsis* had different regulatory networks between AdWRKY40 and its orthologs. AtWRKYs activate *COR47*, *RD21*, and *RD29A* (Song et al., 2010; Hu et al., 2013). AdWRKY40 control *COR47* by potential binding to the W-box element. AdWRKY40 indirectly regulates *RD21* and *RD29A* because they lack the W-box element.

Protein–protein analyses are different from transcriptional regulation. To the best of our knowledge, no study has shown that the abovementioned proteins interact with WRKYs in *Arabidopsis* under drought stress. However, protein–protein interaction has been predicted based on *Arabidopsis* protein in *A. duranensis*. This study found that the NAC transcription factor interacts with WRKY involved in the drought stress response of *A. duranensis*. MaNAC5 interacts with MaWRKY1 and MaWRKY2 to activate *PR1-1*, *PR2*, *PR10c*, and *CHIL1* expressions in response to *Colletotrichum musae* infection in bananas (Shan et al., 2016). Moreover, the CitNAC62-CitWRKY1 interaction enhances *CitAco3* expression, thus decreasing citric acid content (Li et al., 2017). We hypothesize that AdWRKY40 and AdNAC interaction can control *RD21* and *RD29A* expression. However, more experimental tests are needed to verify the hypothesis. Therefore, this study provides a model for studying gene function and a basis for revealing WRKY regulatory networks in *A. duranensis* under drought stress.

## Conclusion

**Figure 8:**
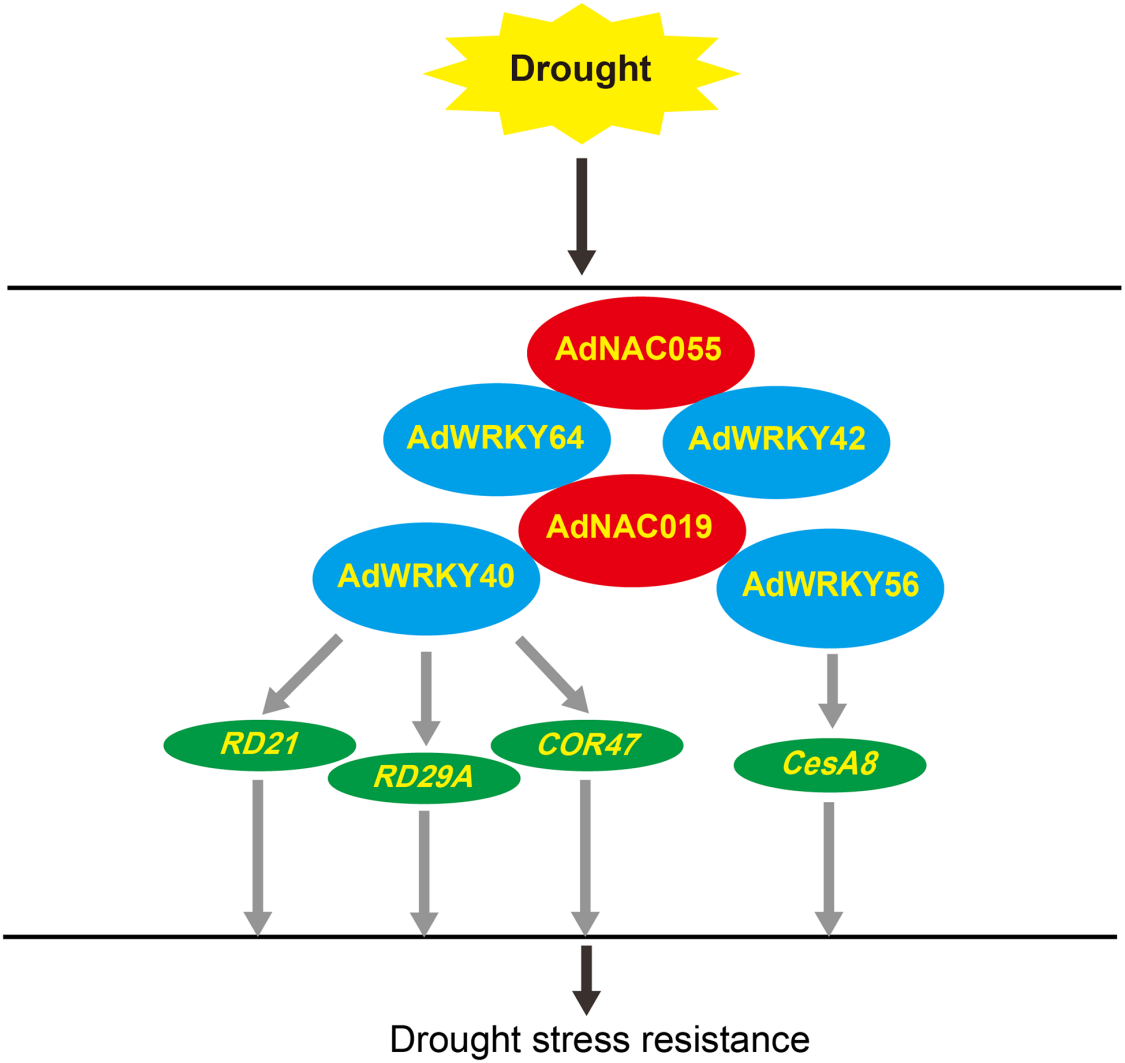
AdWRKYs regulatory networks under drought stress. The gray arrow indicates hypothesis but no experimental test.

This study showed that *AdWRKY18*, *AdWRKY40*, *AdWRKY42*, *AdWRKY56*, and *AdWRKY64* were differentially expressed under drought stress, but various regulatory networks were formed among the five *AdWRKYs*. *AdWRKY18* was excluded from regulatory network analyses because it did not have the same differential expression pattern at two time-points. AdWRKY40 potentially regulates *COR47* by binding the W-box element and indirectly regulates *RD21* and *RD29A* under drought stress. AdWRKY56 controlled the *CesA8* expression under drought stress (Figure 8). Protein–protein interaction results showed that AdNAC019 interacted with AdWRKY40, AdWRKY42, AdWRKY56, and AdWRKY64 under drought stress AdNAC055 interacted with AdWRKY42 and AdWRKY64 (Figure 8).

## Data Availability Statement

Publicly available datasets were analyzed in this study. This data can be found at: https://www.ncbi.nlm.nih.gov/, JZ390113 to JZ390862.

## Author Contributions

HS conceived and designed this research, analyzed data, and wrote the manuscript. YZ and PD analyzed data. PD, FX, XZ, and HS evaluated and revised the manuscript. All authors contributed to the article and approved the submitted version.

## Funding

This study was funded by the Natural Science Foundation of Shandong Province, China (ZR2019QC017), Start-up Foundation for High Talents of Qingdao Agricultural University (no. 665/1120012), the first Class Grassland Science Discipline Program of Shandong Province, China, and Shandong Modern Agricultural Industrial and Technical System (SDAIT-23-01).

## Conflict of Interest

The authors declare that the research was conducted in the absence of any commercial or financial relationships that could be construed as a potential conflict of interest.

## Publisher’s Note

All claims expressed in this article are solely those of the authors and do not necessarily represent those of their affiliated organizations, or those of the publisher, the editors and the reviewers. Any product that may be evaluated in this article, or claim that may be made by its manufacturer, is not guaranteed or endorsed by the publisher.

## Footnotes

^1^ https://string-db.org

## Supplementary Material

Supplementary Table S1 Quantitative real-time PCR primers.

Supplementary Table S2 W-box element and subcellular localization of downstream genes.

Supplementary Table S3 Protein–protein interaction sequences.

Supplementary Table S4 Common interaction between proteins and AdWRKYs.

Supplementary Table S5 Specific interaction between proteins and AdWRKYs.

Supplementary Figure S1 The maximum likelihood tree constructed using WRKY domains.

Supplementary Figure S2 The maximum likelihood tree constructed using full-length WRKY proteins.
