# Supplementary figures and images for: WRKY Genes Improve Drought Tolerance in Arachis duranensis
Source: Front Plant Sci. 2022 May 26;13:910408. doi: 10.3389/fpls.2022.910408 (PMC9199494; doi:10.3389/fpls.2022.910408)

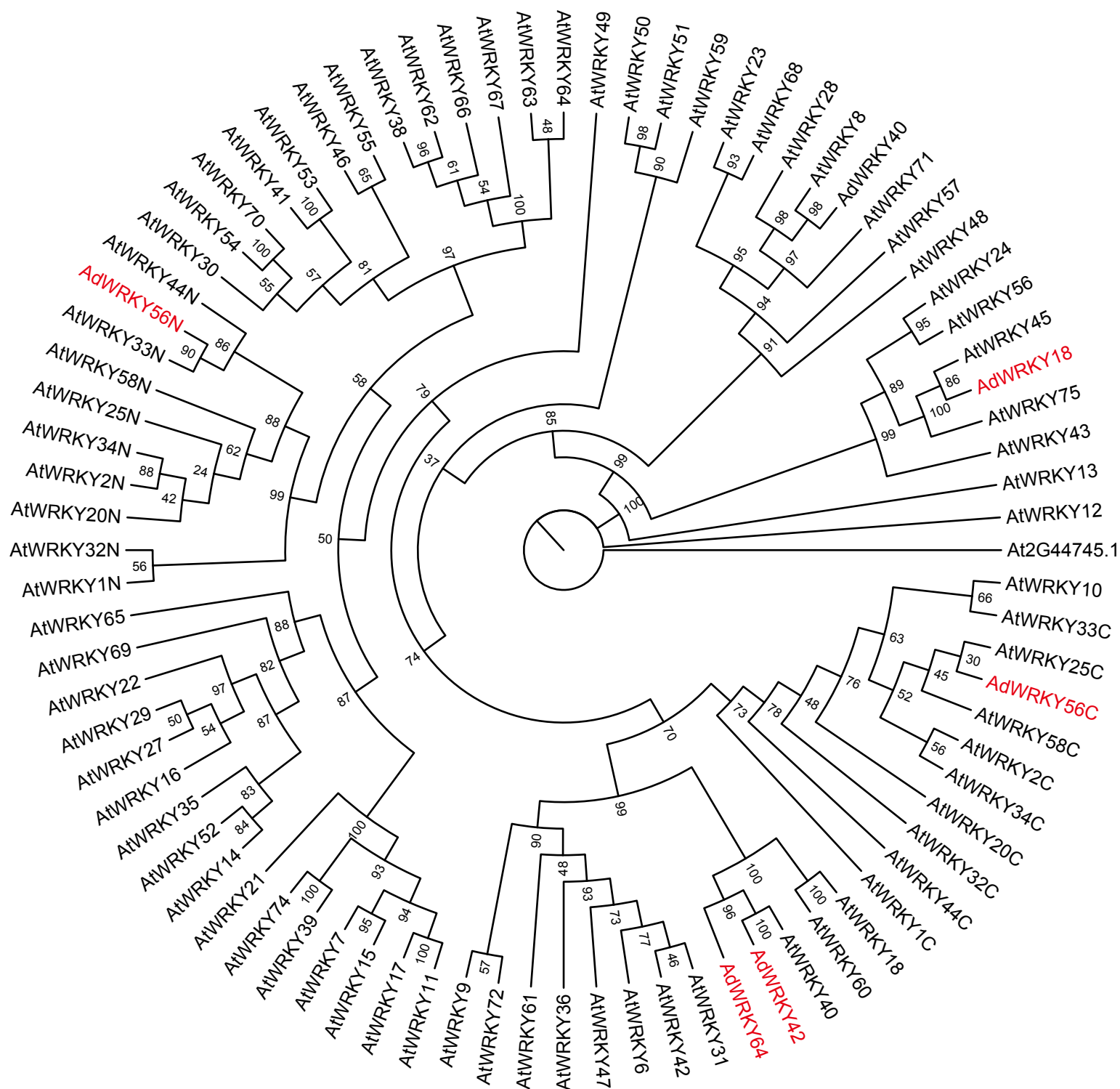

Supplement: Supplementary Figure S1 — The maximum likelihood tree constructed using WRKY domains. [file Data_Sheet_1.PDF]

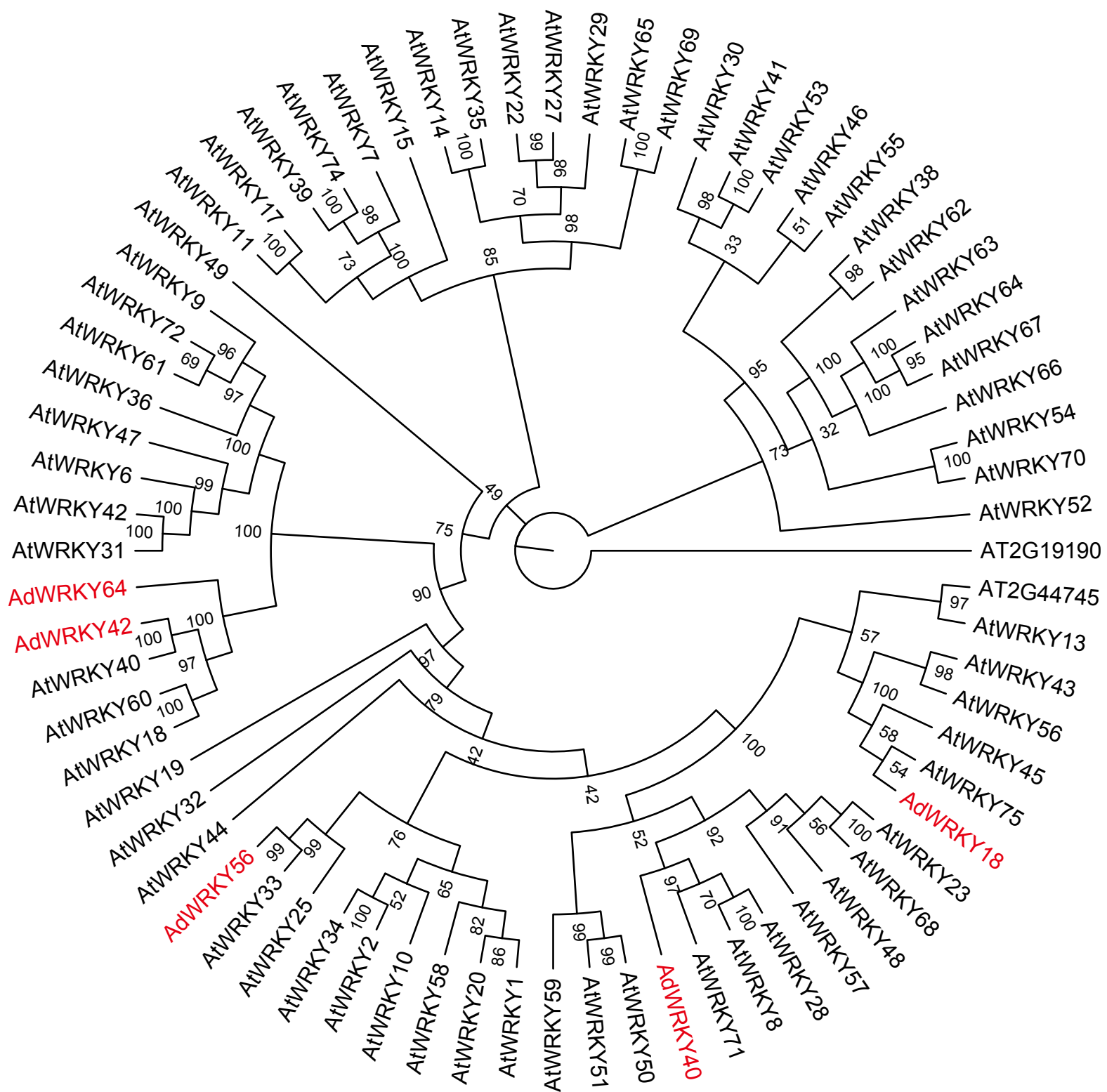

Supplement: Supplementary Figure S2 — The maximum likelihood tree constructed using full-length WRKY proteins. [file Data_Sheet_2.PDF]
